# Preoperative predictive factors associated with severe postdischarge pain after ambulatory gynaecological laparoscopy: a prospective cohort study^☆^

**DOI:** 10.1016/j.bjao.2026.100533

**Published:** 2026-02-27

**Authors:** Mi Stjernberg, Johan Ræder, Milada Hagen, Berit Taraldsen Valeberg, Marlin Comelon

**Affiliations:** 1 Department of Research and Development, Division of Emergencies and Critical Care, Oslo University Hospital, Oslo, Norway; 2 Institute of Clinical Medicine, Faculty of Medicine, University of Oslo, Oslo, Norway; 3 Department of Anaesthesiology and Intensive Care, Division of Emergencies and Critical Care, Oslo University Hospital, Oslo, Norway; 4 Department of Nursing and Health Promotion, Faculty of Health Sciences, Oslo Metropolitan University, Oslo, Norway

**Keywords:** ambulatory anaesthesia, ambulatory surgery, gynaecological laparoscopy, multimodal analgesia, postdischarge pain, postoperative pain, pain predictors, risk factors

## Abstract

**Background:**

Ambulatory surgery patients are at risk of postoperative pain despite modern multimodal pain regimens, and these patients are particularly vulnerable to postdischarge pain due to limited access to medical support. This study explored 24 previously suggested preoperative predictive factors with the primary aim to determine which factors are associated with severe postdischarge pain in women undergoing ambulatory gynaecological laparoscopy with standardised anaesthesia and multimodal pain prophylaxis and treatment.

**Methods:**

In this observational prospective single-centre cohort study, women undergoing ambulatory gynaecological laparoscopy were enrolled. All patients received TIVA (propofol/remifentanil) and multimodal pain prophylaxis including paracetamol, nonsteroidal anti-inflammatory drugs, glucocorticoids, and local anaesthesia. Data were collected before surgery, upon discharge from the PACU, and on postoperative day 1. Univariable and multivariable logistic regression analyses were conducted.

**Results:**

In the final analyses, 439 patients were included. Four preoperative predictive factors were independently associated with severe postdischarge pain (odds ratio, 95% confidence interval): younger age: 0.95, 0.92–0.97, *P*<0.001; preoperative pain: 2.53, 1.58–4.05, *P*<0.001; preoperative opioid use: 1.89, 1.12–3.20, *P*=0.018; and expecting severe postoperative pain: 1.20, 1.07–1.33, *P*=0.001. Severe pain was experienced by 20.7% of the patients during PACU stay and by 42.4% after discharge. Severe pain resulted in hospital admission in 2.3% of the patients and need of health care contact in 3.9%.

**Conclusions:**

Postdischarge pain remains a significant problem after ambulatory gynaecological laparoscopies despite adherence to guidelines for analgesic management. Preoperative predictive factors for severe postdischarge pain were younger age, preoperative pain, use of opioids before surgery and expecting severe postoperative pain. Preoperative identification of at-risk patients may allow for individualised pain prophylaxis and treatment.

**Clinical trial registration:**

NCT05050708.

Studies have indicated that moderate to severe postoperative pain occurs in 20–40% of patients.^1^, ^2^, ^3^ In ambulatory surgery, the patients are extra vulnerable to problems and discomfort related to postdischarge pain because of limited access to professional medical advice and rescue analgesics.^4^ Previous research suggests that the most severe pain is reported during the first day after surgery.^5^ Identifying at-risk patients before surgery would enable us to customise additional prophylactic analgesic interventions for better postoperative pain treatment.

Many studies have identified possible predictors of postoperative pain related to patient characteristics,^2^^,^^5^, ^6^, ^7^, ^8^, ^9^ psychological factors,^5^, ^6^, ^7^, ^8^, ^9^, ^10^ and the surgical procedure.^5^^,^^7^ However, the literature regarding many of these possible predictors is inconsistent.^9^ Most earlier studies have explored only a limited number of predictive factors simultaneously, not considering the potential confounding influence from other co-existing factors. Accordingly, we sought to include a large set (*n*=24) of previously suggested preoperative possible predictive factors concomitantly, to determine which factors are independently and most strongly associated with severe pain after standardised surgery.

The aim of this study was to identify which of several previously suggested preoperative patient-related possible predictive factors are associated with severe postdischarge pain in women undergoing ambulatory gynaecological laparoscopy with standardised anaesthesia and multimodal pain prophylaxis and treatment.

The secondary aims were to estimate the prevalence of severe postoperative pain in the patient sample, during the PACU stay, after discharge until 24 h after surgery, and on postoperative day 1 (POD1).

## Methods

### Ethics

The research project was approved by the Regional Committee for Medical and Health Research Ethics of South-East Norway (Reg. No. 218704) and registered in ClinicalTrials.gov (Reg. No. NCT05050708), with pre-planned outcomes including postoperative pain and nausea. This manuscript reports analyses of postsurgical pain only; nausea results will be presented separately. Written informed consent was obtained from patients willing to participate.

### Study design and participants

In this observational prospective cohort study, we recruited women scheduled for ambulatory gynaecological laparoscopy at Oslo University Hospital, Norway, between August 2021 and June 2022.

### Inclusion criteria

The patient had to be ≥18 yr, speak Norwegian, and have no contraindications to the standard intraoperative pain prophylaxis medication. Patients with severe psychiatric disorder, cognitive dysfunction, or drug use disorder were not included.

### Anaesthesia and intraoperative multimodal pain prophylaxis

All patients received TIVA with target-controlled propofol and remifentanil infusions. Pain prophylaxis consisted of i.v. paracetamol 1000 mg, ketorolac 30 mg or parecoxib 40 mg, and dexamethasone 8 mg. All patients received fentanyl 50–100 mcg i.v. and bupivacaine 40–50 mg for surgical site infiltration at the end of surgery.

Additional intraoperative i.v. multimodal nausea prophylaxis was provided by droperidol 1.25 mg and ondansetron 4 mg.

### In-hospital analgesics

Postoperative breakthrough pain was treated with i.v. fentanyl 25–50 mcg or i.v. oxycodone 2–5 mg. A single-dose i.v. ketorolac 15 mg or parecoxib 20 mg could be repeated 3 h after the intraoperative dose. Paracetamol 1000 mg p.o. was repeated after 4–6 h. Other rescue analgesics used were oral immediate-release oxycodone 5 mg, modified-release oxycodone 5–10 mg, tramadol 50 mg, and i.v. or oral clonidine 75 mcg. Patients had to be adequately pain-relieved and free from nausea to be discharged home.

### Postdischarge analgesic regime

All patients received oral and written instructions on how to administer p.o. paracetamol 1000 mg and ibuprofen 400 mg four times daily, after discharge. To manage breakthrough pain, the patients were provided with an in-house supply of four capsules of immediate-release oxycodone 5 mg intended for stronger pain, and six capsules of tramadol 50 mg for moderate pain. No additional opioids were prescribed.

### Data collection

Two weeks before the planned surgery, study information and an invitation to participate were sent to eligible patients. Data were collected on three different occasions: before surgery, after surgery upon discharge from the PACU, and on POD1. The data were registered in a web questionnaire solution (Nettskjema) and stored at the University of Oslo’s Service for Sensitive Data.

### Preoperative data collection

A text message containing a link to a questionnaire was sent 1–2 days before surgery. The questionnaire included self-reported data on patient characteristics, preoperative pain (i.e. any location or related to upcoming surgery), average preoperative pain the week before surgery, and preoperative analgesics use within the past 4 weeks (Table 1). Additionally, six validated questionnaires addressing psychological and physiological measures with possible impact on postoperative pain were included: Pain Catastrophizing Scale (PCS),^12^ Hospital Anxiety and Depression Scale (HADS),^13^ General Self-Efficacy Scale (GSE),^14^^,^^15^ Pain Sensitivity Questionnaire (PSQ),^16^ Bergen Insomnia Scale (BIS),^17^ and Christensen Fatigue Scale (CFS)^18^ (Table 2). All questionnaires have previously been used in Norwegian study samples.^17^^,^^22^, ^23^, ^24^, ^25^, ^26^ After a thorough review of the literature, including relevant systematic reviews, together with the research team’s long experience with ambulatory surgery, anaesthesia, and perioperative medicine, a total of 24 preoperative possible predictive factors were selected. The 24 preoperative factors assumed relevant for severe pain after surgery are listed in Table 3.

**Table 1:** Patient characteristics of the total sample (*n*=439). NRS categories according to Breivik and colleagues.^11^ ∗Continuous variable. IQR, interquartile range; NRS, numeric rating scale.

Variables	*n* (%)	Mean (sd)/median (IQR)
Age (yr)∗ (range 18–77)		30 (25–38)
Height (cm)∗		167.8 (6.2)
Weight (kg)∗		68.2 (12.5)
BMI (kg m^−2^)∗		24.3 (4.4)
ASA physical status		
1	257 (58.5)	
2	176 (40.1)	
3	6 (1.4)	
Single-person household		
No	332 (75.6)	
Yes	107 (24.4)	
Education		
Upper secondary school/other	157 (35.8)	
College/university <4 yr	131 (29.8)	
College/university ≥4 yr	151 (34.4)	
Employment status		
Employed	390 (89.0)	
Unemployed or >50% on sick leave	48 (11.0)	
Financial concerns		
No	263 (60.2)	
Yes	174 (39.8)	
Smoking		
No	384 (88.3)	
Yes, daily/occasionally	51 (11.7)	
Previous abdominal surgery		
No	279 (63.8)	
Yes	158 (36.2)	
Preoperative pain (any location)		
No	190 (43.5)	
Yes	247 (56.5)	
Average pain week pre-surgery (NRS 0–10)∗		3 (1–5)
NRS 0	82 (19.0)	
NRS 1–3	144 (33.3)	
NRS 4–6	138 (32.0)	
NRS 7–10	68 (15.7)	
Expected postoperative pain (NRS 0–10)∗		5.4 (2.2)
NRS 0	10 (2.3)	
NRS 1–3	72 (16.6)	
NRS 4–6	221 (50.9)	
NRS 7–10	131 (30.2)	
Worst menstrual pain (NRS 0–10)∗		7 (3–9)
NRS 0	93 (21.2)	
NRS 1–3	21 (4.8)	
NRS 4–6	71 (16.2)	
NRS 7–10	253 (57.8)	
Preoperative non-opioid analgesics past 4 weeks		
No	80 (18.3)	
Yes	358 (81.7)	
Preoperative opioids past 4 weeks		
Daily	4 (0.9)	
Weekly	28 (6.5)	
Less than weekly	69 (16.0)	
Not applicable	331 (76.6)

**Table 2:** Preoperative questionnaires and comparison between Group NS (NRS 0–6) and Group S (NRS 7–10) (*n*=439). The scores from the questionnaires are used as continuous variables when comparing groups (Group NS *vs* Group S). In addition, the distribution is shown as categories using cut-off scores, with references indicated. HADS, Hospital Anxiety and Depression Scale; HADS-A, Hospital Anxiety and Depression Scale—Anxiety; HADS-D: Hospital Anxiety and Depression Scale—Depression; IQR, interquartile range; NRS, numeric rating scale. ∗Comparison between Group NS and Group S. **^†^**Continuous variable.

Variables	Total sample		Group NS (*n*=253)	Group S (*n*=186)	*P*-value∗
	*n* (%)	Mean (sd)/median (IQR)			
General Self-Efficacy^†^ (10–40)		30.4 (5.4)	30.6 (5.5)	30.0 (5.2)	0.266
<Cut-off 30^15^	185 (42.8)		100 (40.5)	85 (45.9)	
≥Cut-off 30^15^	247 (57.2)		147 (59.5)	100 (54.1)	
Pain Catastrophizing Scale—Total^†^ (0–52)		15.4 (9.6)	13.7 (8.9)	17.8 (10)	<0.001
<Cut-off 30^19^	397 (90.8)		238 (94.4)	159 (85.9)	
≥Cut-off 30^19^	40 (9.2)		14 (5.6)	26 (14.1)	
Pain Sensitivity Questionnaire—Total^†^ (0–10)		3.0 (1.2)	2.8 (1.1)	3.1 (1.3)	0.016
HADS—Total^†^ (0–42)		11.0 (6.5)	9.6 (6.3)	13.0 (6.4)	<0.001
<Cut-off 19^20^	374 (85.6)		229 (90.5)	145 (78.8)	
≥Cut-off 19^20^	63 (14.4)		24 (9.5)	39 (21.2)	
HADS-A^†^ (0–21)		7.5 (4.1)	6.6 (4.1)	8.7 (3.8)	<0.001
HADS-A <cut-off 8^21^	223 (51.0)		152 (60.1)	71 (38.6)	
HADS-A ≥cut-off 8^21^	214 (49.0)		101 (39.9)	113 (61.4)	
HADS-D^†^ (0–21)		3 (1–6)	2 (1–5)	4 (1–7)	<0.001
HADS-D <cut-off 8^21^	376 (86.0)		230 (90.9)	146 (79.3)	
HADS-D ≥cut-off 8^21^	61 (14.0)		23 (9.1)	38 (20.7)	
Bergen Insomnia Scale^†^ (0–42)		16.4 (9.7)	14.5 (9.4)	19.1 (9.5)	<0.001
No insomnia^17^	164 (37.5)		112 (44.3)	52 (28.3)	
Insomnia^17^	273 (62.5)		141 (55.7)	132 (71.7)	
Christensen Fatigue Scale^†^ (1–10)		4 (2–6)	3 (2–5)	4 (3–6)	<0.001
<Cut-off 6^22^	314 (73.2)		191 (76.7)	123 (68.3)	
≥Cut-off 6^22^	115 (26.8)		58 (23.3)	57 (31.7)

**Table 3:** Univariable and multivariable logistic regression analyses of preoperative possible predictive factors associated with worst postdischarge pain. Dependent variable dichotomised into NRS 0–6/NRS 7–10. Independent variables with a *P*-value <0.1 in the univariable analysis were entered in the multivariable regression model (*n*=439). CI, confidence interval; NRS, numeric rating scale; HADS-A, Hospital Anxiety and Depression Scale—Anxiety; HADS-D, Hospital Anxiety and Depression Scale—Depression; OR, odds ratio; Ref., reference value. ∗Continuous variable. ^†^Variable entered in the multivariable regression model.

Variables	Univariable analysis			Multivariable analysis		
	OR	95% CI	*P*-value	OR	95% CI	*P*-value
Age (yr)∗ (Ref. 18)	0.93	0.91–0.95	<0.001^†^	0.95	0.92–0.97	<0.001
Height (cm)∗ (Ref. 144)	0.98	0.95–1.01	0.113			
Weight (kg)∗ (Ref. 42)	0.99	0.98–1.01	0.446			
BMI∗ (Ref. 14)	1.00	0.95–1.04	0.875			
ASA physical status						
1 (Ref.)	1					
2	1.08	0.73–1.60	0.688			
3	2.85	0.51–15.84	0.232			
Single-person household						
Yes (Ref.)	1					
No	1.39	0.88–2.17	0.155			
Education						
Upper secondary school (Ref.)	1					
College/university <4 yr	0.61	0.38–0.97	0.038			
College/university ≥4 yr	0.41	0.26–0.64	<0.001^†^			
Employment status						
Unemployed or >50% on sick leave (Ref.)	1					
Employed	0.58	0.32–1.06	0.079^†^			
Financial concerns						
No (Ref.)	1					
Yes	1.42	0.96–2.09	0.078^†^			
Smoking						
No (Ref.)	1					
Yes	0.95	0.52–1.72	0.863			
Previous abdominal surgery						
No (Ref.)	1					
Yes	1.01	0.68–1.49	0.982			
Preoperative pain (any location)						
No (Ref.)	1					
Yes	3.23	2.15–4.86	<0.001^†^	2.53	1.58–4.05	<0.001
Average pain week pre-surgery∗ (Ref. NRS 0)	1.30	1.20–1.40	<0.001^†^			
Expected postoperative pain∗ (Ref. NRS 0)	1.32	1.20–1.46	<0.001^†^	1.20	1.07–1.33	0.001
Worst menstrual pain						
0–6 (Ref.)	1					
7–10	3.68	2.43–5.58	<0.001^†^	1.45	0.87–2.41	0.153
Preoperative non-opioid analgesics						
No (Ref.)	1					
Yes	2.78	1.59–4.84	<0.001^†^			
Preoperative opioids						
No (Ref.)	1					
Yes	2.58	1.63–4.07	<0.001^†^	1.89	1.12–3.20	0.018
Questionnaires						
General Self-Efficacy∗ (Ref. 10)	0.98	0.95–1.02	0.266			
Pain Catastrophizing Scale—Total∗ (Ref. 0)	1.05	1.03–1.07	<0.001^†^	0.98	0.96–1.01	0.265
Pain Sensitivity Questionnaire—Total∗ (Ref. 0)	1.21	1.04–1.42	0.017^†^			
HADS-A∗ (Ref. 0)	1.13	1.08–1.19	<0.001^†^	1.06	0.99–1.13	0.080
HADS-D∗ (Ref. 0)	1.13	1.07–1.20	<0.001^†^			
Bergen Insomnia Scale∗ (Ref. 0)	1.05	1.03–1.07	<0.001^†^			
Christensen Fatigue Scale∗ (Ref. 1)	1.17	1.07–1.27	<0.001^†^			

### Postoperative data collection

Upon discharge, patients reported ‘worst postoperative pain’ experienced during the PACU stay. Type of surgery, surgery and anaesthesia duration, time spent in the PACU, and pain medication administered postoperatively before discharge were registered.

### Postdischarge data collection

The patients were telephone-interviewed on POD1, approximately 24 h after surgery, and asked to rate present pain at rest and during mobilisation, average pain on POD1, and worst pain experienced since discharge (i.e. in any situation) (Table 4). Analgesic consumption from discharge until 24 h after surgery and unscheduled contacts with health care facilities and hospital admittance were recorded. If admitted or readmitted to hospital, analgesic consumption was collected from the patient’s medical records.

**Table 4:** Postoperative pain assessments during PACU stay and on postoperative day 1, using the 11-point numeric rating scale. Median OMEQ doses in the PACU, postdischarge, and total (PACU+postdischarge) are presented. NRS is used as a continuous variable when comparing groups (Group NS *vs* Group S). In addition, the NRS is categorised using published cut-off scores, as defined by Breivik and colleagues.^11^ The Norwegian Directorate of Health’s opioid calculator was used for calculations of OMEQs. IQR, interquartile range; NRS, numeric rating scale; OMEQ, oral morphine equivalents. ∗Comparison between Group NS and Group S. ^†^Continuous variable.

Variables	Total sample (*n*=439)		Group NS (NRS 0–6) (*n*=253)		Group S (NRS 7–10) (*n*=186)		*P*-value∗
	*n* (%)	Mean (sd)	*n* (%)	Mean (sd)	*n* (%)	Mean (sd)	
Worst pain in PACU^†^ (0–10)		4.6 (2.2)		3.8 (2.0)		5.6 (2.0)	<0.001
NRS 0–3	137 (31.5)		110 (44.2)		27 (14.5)		
NRS 4–6	208 (47.8)		116 (46.6)		92 (49.5)		
NRS 7–10	90 (20.7)		23 (9.2)		67 (36.0)		
OMEQ in PACU^†^, median (IQR)	369 (84.1)	10.5 (7.5–15.0)	198 (78.3)	7.5 (7.5–15.0)	171 (91.9)	12.0 (7.5–16.5)	<0.001
Postoperative day 1							
Pain at rest^†^ (0–10)		3.2 (2.0)		2.2 (1.4)		4.5 (1.9)	<0.001
NRS 0–3	252 (57.4)		202 (79.8)		50 (26.9)		
NRS 4–6	159 (36.2)		51 (20.2)		108 (58.1)		
NRS 7–10	28 (6.4)		0		28 (15.0)		
Pain during activity^†^ (0–10)		4.8 (2.2)		3.5 (1.5)		6.5 (1.8)	<0.001
NRS 0–3	130 (29.6)		122 (48.2)		8 (4.3)		
NRS 4–6	206 (46.9)		131 (51.8)		75 (40.3)		
NRS 7–10	103 (23.5)		0		103 (55.4)		
Average pain^†^ (0–10)		3.9 (1.8)		2.8 (1.2)		5.4 (1.4)	<0.001
NRS 0–3	195 (44.4)		181 (71.5)		14 (7.5)		
NRS 4–6	209 (47.6)		72 (28.5)		137 (73.7)		
NRS 7–10	35 (8.0)		0		35 (18.8)		
Worst pain since discharge^†^ (0–10)		5.9 (2.3)		4.3 (1.5)		8.1 (1.0)	<0.001
NRS 0–3	78 (17.8)		78 (30.8)		0		
NRS 4–6	175 (39.8)		175 (69.2)		0		
NRS 7–10	186 (42.4)		0		186 (100)		
OMEQ postdischarge^†^, median (IQR)	277 (63.1)	7.5 (0.0–15.0)	130 (51.4)	7.5 (0.0–15.0)	147 (79.0)	15.0 (7.5–22.5)	<0.001
OMEQ total (PACU+postdischarge)^†^, Median (IQR)	399 (90.9)	19.5 (7.5–30.0)	217 (85.8)	15.0 (7.5–22.5)	182 (97.8)	25.5 (15.0–36.4)	<0.001

### Outcome measures

Pain was measured on the 11-point numeric rating scale (NRS), 0 being ‘No pain at all’ and 10 being ‘Worst imaginable pain’. Severe pain was defined as NRS 7–10.^2^^,^^5^^,^^11^ Postoperative and postdischarge pain measures were related to the surgical area in the abdomen.

To identify patients with severe postdischarge pain, we used the variable ‘Worst pain since discharge’ to categorise the patients into two groups: patients with non-severe pain (Group NS), NRS 0–6, and patients with severe pain (Group S), NRS 7–10. Also, in the logistic regression models, ‘Worst pain since discharge’ was used as the dependent variable, dichotomised into NRS 0–6 and 7–10.

### Statistical analyses

#### Sample size consideration

We planned to perform a multivariable logistic regression and anticipated that at least 20% of patients would experience severe pain. We originally aimed to include 300 patients, thereby having statistical power to fit a model with up to six covariates using a rule of thumb of at least 10 individuals in the smallest category of the outcome per covariate.

Patients with data registrations at all three time points were included in the analyses. Continuous variables are presented as mean (sd), if normally distributed, or as median (interquartile range [IQR]) if skewed. Categorical data are presented as counts and percentages.

To compare groups (Group NS and Group S), independent-samples *t*-test was used on normally distributed continuous data, Mann–Whitney *U*-test on continuous data with skewed distribution, and χ^2^ test on pairs of categorical variables (Tables 2 and 4, Supplementary Table 1).

We used univariable logistic regression to identify variables associated with severe postdischarge pain at a *P*<0.1 level. Possible predictors with *P*<0.1 were subsequently included in a multivariable logistic regression model. The final model was derived using backward conditional selection process. Results are reported as odds ratios (ORs) and 95% confidence intervals (95% CIs). *P*<0.05 was considered statistically significant (Table 3).

IBM SPSS Statistics for Windows, version 29.0 (IBM Corp., Armonk, NY, USA) was used for statistical analyses.

## Results

**Fig 1:**
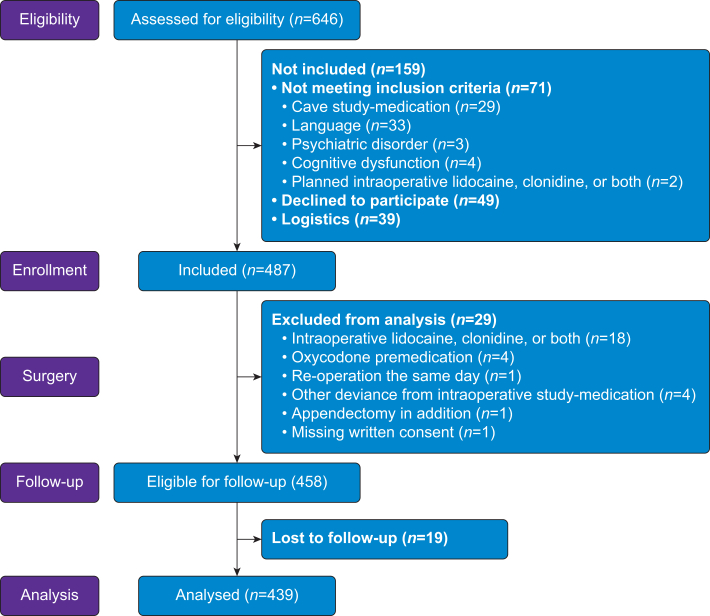
Flow diagram.

Of 646 eligible patients, 487 were enrolled in the study, and 439 patients with data registrations at all three time points were included in the final analyses (Fig. 1).

### Patients’ preoperative characteristics

In the total sample, the median age was 30 yr. Most patients were classified as ASA 1–2 (98.6%), employed (89.0%), non-smokers (88.3%) and had a mean BMI of 24.3. Preoperative pain was present in 56.5%, and severe dysmenorrhea was reported by 57.8%. Within the past 4 weeks before surgery, analgesics were used by 81.7%, although the majority were opioid-naïve (76.6%). Approximately one-third of the patients expected severe postoperative pain (Table 1).

Patients in Group S (*n*=186) were significantly younger than those in Group NS (*n*=253) and had lower education, higher prevalence of preoperative pain, more intense pain, more severe dysmenorrhea, more frequent use of preoperative analgesics (including opioids), and expected stronger postoperative pain (Supplementary Table 1). Group S also scored significantly higher on the composite scores for all questionnaires, except for GSE (*P*=0.266) (Table 2).

### Intraoperative characteristics

The median surgery duration was 38 min. Among the 11 surgical procedures performed, laparoscopic excision of endometriosis was the most common (48.1%) (Supplementary Table 2) and occurred significantly more frequently in Group S *vs* Group NS (61% and 38%, respectively; *P*<0.001). No significant difference in surgery duration was observed between the two groups (*P*=0.278) (data not shown).

### Postoperative pain and analgesic consumption

In total, severe pain in the PACU was reported by 20.7%, and Group S reported higher pain scores three times more often than Group NS (*P*<0.001) (Table 4). The median PACU stay was 171 min (Supplementary Table 2). Forty-three patients received clonidine i.v. during the PACU stay, of whom 28 belonged to Group S (data not shown). The oral morphine equivalents (OMEQ) median doses in the PACU were 12.0 mg (IQR, 7.5–16.5) and 7.5 mg (IQR, 7.5–15.0) in Group S and NS, respectively (*P*<0.001) (Table 4).

From discharge until 24 h after surgery, 42.4% of the patients experienced severe pain. On POD1, 6.4% had severe pain at rest, and 8% reported their average pain to be severe. The differences between Group S and NS were statistically significant for all pain variables. After discharge, 99.3% of the patients used analgesics at some point, of whom 62.6% took opioids (data not shown), primarily immediate-release oxycodone. Most patients used paracetamol and ibuprofen, and further details on postdischarge analgesia are presented in Supplementary Table 3. Postdischarge OMEQ median dose was 15.0 mg (IQR, 7.5–22.5) in Group S and 7.5 mg (IQR, 0.0–15.0) in Group NS (*P*<0.001) (Table 4).

### Planned hospital discharge and unscheduled contacts

Approximately 95% of the patients were discharged home as planned. Of the 5% admitted to hospital, 10 patients (2.3%) were admitted because of severe pain. Nineteen (4.3%) individual contacts with health care facilities occurred within 24 h after surgery. Seventeen of these contacts were related to postsurgical pain, of which 15 were Group S patients (data not shown).

### Preoperative possible predictive factors associated with severe postdischarge pain

In the univariable logistic regression analysis, 16 of the 24 preoperative possible predictive factors were associated with severe postdischarge pain at a significance level of *P*<0.1. In the final multivariable model, only four of these 16 predictive factors remained independently associated with severe postdischarge pain: age, preoperative pain, opioid use, and expecting severe postoperative pain. For each additional year of age, the odds for severe pain decreased by 5% (OR, 95% CI: 0.95, 0.92–0.97; *P*<0.001), indicating less pain with increased age. Patients with pre-existing pain were 2.5 times more likely to report severe postdischarge pain (OR, 95% CI: 2.53, 1.58–4.05; *P*<0.001). Patients who used opioids were almost 90% more likely to have severe postdischarge pain (OR, 95% CI: 1.89, 1.12–3.20; *P*=0.018). Pain expectation was measured on a NRS (0–10), and the odds for severe pain were 20% higher for each incremental step on the NRS (OR, 95% CI: 1.20, 1.07–1.33; *P*=0.001) (Table 3).

Of the six questionnaires, all but GSE were statistically significant in the univariable analysis, but none remained statistically significant in the multivariable model (Table 3).

## Discussion

In this prospective cohort study, we identified four preoperative predictive factors independently associated with severe postdischarge pain until 24 h after ambulatory gynaecological laparoscopic surgery: younger age, preoperative pain, expecting severe postoperative pain, and preoperative opioid use. We also found that postoperative pain remains a significant problem in the immediate postoperative period despite adherence to multimodal guidelines for pain management. Severe pain in the PACU was reported by one-fifth of the patients, and >40% experienced severe pain between discharge and 24 h post-surgery. Statistically significant differences were observed in both postoperative and postdischarge pain levels, and opioid consumption, all in disfavour of Group S. This underscores the importance of identifying individuals at risk of severe pain after surgery, in order to provide tailored pain prophylaxis and opioid-sparing strategies.

Consistent with previous literature, younger patients reported more severe pain during the first 24 h after surgery,^2^^,^^3^^,^^5^^,^^7^, ^8^, ^9^ which may partly be attributable to age-related changes in pain perception.^27^ The odds for severe pain in our sample were 0.95 per year increase; that is, when compared with a 50-yr-old, the odds for severe pain are 1.67, 2.72, and 3.49 higher for patients aged 40, 30, and 25 yrs, respectively.

Patients with pre-existing pain, whether related to the upcoming surgery site or elsewhere, were more likely to experience severe postdischarge pain. Ongoing nociceptive input and central sensitisation may contribute to heightened pain responses after surgery, and our results support previous studies on preoperative pain as a predictor for postoperative pain.^2^^,^^3^^,^^7^, ^8^, ^9^^,^^28^

Preoperative opioid use is known as a significant indicator of postoperative pain severity and was independently associated with severe postdischarge pain in our patients, unlike the use of non-opioid analgesics. Preoperative opioids may cause opioid tolerance and opioid-induced hyperalgesia and have previously been identified as a predictive factor in studies including mixed surgical inpatients^5^ and orthopaedic patients.^29^ Many studies refer to ‘preoperative analgesic consumption’ without distinguishing between opioids and non-opioids, which limits the understanding of opioids as an independent risk factor for postoperative pain. In our study, >75% of the patients were opioid-naïve, and few patients had used opioids daily.

Pain expectation may affect acute postoperative pain intensity^9^^,^^10^^,^^28^ and emerged as a significant predictive factor for severe pain in our study. Stessel and colleagues^28^ found that patients who expected postoperative pain were more likely to experience moderate to severe pain even on POD4 after ambulatory surgery. In our study, nearly one-third of the patients expected severe pain after surgery.

As the study focused on the surgical speciality gynaecology, our patient sample included only women, which may have contributed to the high pain prevalence. Yang and colleagues^8^ reported that women had 30% increased odds for postoperative pain compared with men. Numerous studies have found women to be more prone to experience moderate to severe postoperative pain,^2^^,^^5^^,^^8^^,^^30^, ^31^, ^32^ even though there is conflicting evidence.^6^^,^^7^ Gynaecological laparoscopy is considered to be minimally invasive surgery, but is still associated with moderate to severe postoperative pain,^1^^,^^3^^,^^5^^,^^33^ despite modern multimodal treatment (e.g. Procedure Specific Postoperative Pain Management [PROSPECT]), as shown in our study. This underscores the need to identify at-risk patients, and to enhance and individualise pain management in this patient population, using simple measures such as improved information, adjuvant analgesics, or increased analgesic doses when possible. As severe postdischarge pain led to several hospital admissions and re-contacts in our study, it may also be of cost-benefit to address this issue.

Surprisingly, none of the psychological variables in this study remained statistically significant in the multivariable regression analysis, despite previous research identifying them as predictors of postoperative pain.^7^, ^8^, ^9^, ^10^ Even though nearly 50% of our patients reported preoperative anxiety, compared with 9.6% in the general Norwegian population,^34^ our results diverged from the studies by Sobol-Kwapinska and colleagues^10^ or Yang and colleagues,^8^ who identified anxiety as one of several psychological predictors of postoperative pain. However, these systematic reviews and meta-analyses included inpatients within multiple surgical specialities, indicating more invasive surgeries with differing pain regimens which may contribute to the diverging results from our study. Additionally, different instruments were used to measure anxiety.

In our study, several preoperative possible predictive factors associated with increased odds of severe postdischarge pain in the univariable analysis did not remain statistically significant in the multivariable regression model. These factors included educational level, employment status, financial concerns, average preoperative pain intensity, dysmenorrhea severity, preoperative use of non-opioid analgesics, pain catastrophising, pain sensitivity, anxiety, depression, insomnia, and fatigue. Furthermore, previously suggested predictive factors, such as height, weight, BMI, ASA class, living arrangement, smoking, previous abdominal surgery, and self-efficacy, were not found to be significantly associated with severe postdischarge pain, even in the univariable analysis. One possible explanation for not being identified as a predictive factor in our study could be the limited statistical power for these variables owing to a predominance of patients in one category, not allowing for proper comparisons. For instance, the patient sample consisted mainly of healthy individuals (ASA 1–2) and non-smokers with low BMI, and most patients were employed.

Previous systematic reviews and meta-analyses have also typically consisted of single studies assessing a limited number of risk factors, not considering the confounding effect of other concomitant possible predictive factors.^7^^,^^8^^,^^10^ In contrast to most earlier studies, we included a large set of previously reported preoperative possible predictive factors into a multivariable regression model. Thus, we hypothesise that some previously suggested individual predictive factors may not stay statistically significant once adjusted for relevant confounders.

This study has several strengths. The main strength is the inclusion of a large number of previously suggested predictive factors associated with postoperative pain in a single study, using multivariable logistic regression. The prospective design enabled us to explore a wide range of preoperative possible predictive factors of interest. A high number of included patients, combined with standardised anaesthesia and recommended multimodal pain prophylaxis and treatment, may have reduced the effect of other potential confounders. We also aimed to identify patients with severe pain despite an ‘optimised’ standard pain regimen, contrary to previous studies, often with non-standardised regimes. The short data collection time frame for each patient reduced the risk of recall bias. Few patients were lost to follow-up, and missing data in the final analyses was <3%.

There are limitations to our study, consisting of an ambulatory female patient sample. As such, the findings cannot be directly applied to other surgical patient populations, including inpatients, men, older patients, and patients with major surgical procedures. Also, variability in genetic predisposition for pain sensitivity, pharmacokinetics and -dynamics of analgesic drugs, which may influence postoperative pain, are not addressed in the study.

Our findings apply to patients treated with multimodal pain prophylaxis using paracetamol, nonsteroidal anti-inflammatory drugs (NSAIDs), and steroids as recommended in international guidelines. Allergy or medical contraindications are, however, rarely found to restrict use of these medications in gynaecological ambulatory surgery patients. Our findings are also limited to patients under general anaesthesia with remifentanil and small-dose fentanyl at the end of surgery, both possibly adding to opioid-induced side-effects such as hyperalgesia and tolerance, which may influence postoperative pain levels.

Our aim was to explore possible predictive factors that can be identified before surgery. It may be argued that invasiveness and duration of surgery can be predicted. However, this is not always the case in gynaecological procedures, where the extent of surgery may be determined intraoperatively. Therefore, we did not include minor *vs* major surgery in the analyses. Similarly, the presence of endometriosis may be a predictive factor of strong postoperative pain, but the preoperative diagnosis of this condition was not available with sufficient level of detail. Further, we did not include severe postoperative pain during PACU stay as a potential predictive factor of severe postdischarge pain, despite its strong association with the main outcome, as it is not known before surgery. Preoperative pain is a well-established predictor for postoperative pain. We asked the patients whether they had pre-existing pain at the time of surgery (i.e. any pain). Although we could have also asked about chronic pain, including its incidence and duration, this was deemed impractical because of its complexity, in a busy preoperative ambulatory surgery setting.

This study was a part of a larger research project with postoperative pain and nausea as pre-specified primary outcomes. To allow focused analysis and interpretation, this manuscript addresses pain-related outcomes, whereas nausea-related analyses will be reported in a separate publication. Readers should interpret the findings within this context.

We acknowledge that there might be other variables associated with postoperative pain not analysed in our study; however, the variables identified in our study are relatively easy to assess before surgery and might aid clinicians in identifying patients in need of additional pain management.

In conclusion, we found younger age, preoperative pain, expecting severe postoperative pain, and preoperative use of opioids to be predictive factors independently associated with severe postdischarge pain in patients undergoing ambulatory gynaecological laparoscopies under general anaesthesia with propofol and remifentanil, and with multimodal pain prophylaxis and treatment. Postdischarge pain remains a significant problem in these patients, and preoperative identification of at-risk patients in need of additional preventive measures could limit the extent of postoperative pain and potentially reduce healthcare costs.

## Authors’ contributions

Conception and study design: all authors

Acquisition of data: MS

Data analysis and interpretation of data: all authors

Drafting the manuscript: MS, MC, JR, MH

Critical reviewing of the manuscript: all authors

Approved the final version of the manuscript and agree to be accountable for all aspects of the work: all authors

## Funding

Department of Research and Development, Division of Emergencies and Critical Care, Oslo University Hospital, Oslo, Norway.

## Declarations of interest

The authors declare that they have no conflicts of interest.
