# Supplementary material for: Preoperative predictive factors associated with severe postdischarge pain after ambulatory gynaecological laparoscopy: a prospective cohort study
Source: BJA Open. 2026 Feb 27;17:100533. doi: 10.1016/j.bjao.2026.100533 (PMC12964276; doi:10.1016/j.bjao.2026.100533)
Supplement: Multimedia component [file mmc1.docx]

Supplementary Table 1. Comparison between patient characteristics for Group NS (NRS 0-6) and Group S (NRS 7-10) (n= 439).

| **Variables** | **Group NS (NRS 0-6)**  (n=253) | | **Group S (NRS 7-10)**  (n=186) | | **P-value** |
| --- | --- | --- | --- | --- | --- |
|  | **n (%)** | **mean (SD)/ median (IQR)** | **n (%)** | **mean (SD)/ median (IQR)** |  |
| **Age** (yr)* Median (IQR) |  | 34 (26.5-42) |  | 27 (23-33) | <0.001 |
| **Height** (cm)* Mean (SD) |  | 168.2 (6.0) |  | 167.3 (6.4) | 0.112 |
| **Weight** (kg)* Mean (SD) |  | 68.6 (11.9) |  | 67.7 (13.2) | 0.447 |
| **BMI** (kg m^-2)^* Mean (SD) |  | 24.3 (4.2) |  | 24.2 (4.6) | 0.875 |
| **ASA physical status** |  |  |  |  | 0.442 |
| 1 | 151 (59.7) |  | 106 (57.0) |  |  |
| 2 | 100 (39.5) |  | 76 (40.8) |  |  |
| 3 | 2 (0.8) |  | 4 (2.2) |  |  |
| **Single-person household** |  |  |  |  | 0.154 |
| No | 185 (73.1) |  | 147 (79.0) |  |  |
| Yes | 68 (26.9) |  | 39 (21.0) |  |  |
| **Education** |  |  |  |  | <0.001 |
| Upper secondary school/other | 73 (28.9) |  | 84 (45.2) |  |  |
| College/university ˂4 yr | 77 (30.4) |  | 54 (29.0) |  |  |
| College/university ≥4 yr | 103 (40.7) |  | 48 (25.8) |  |  |
| **Employment status** |  |  |  |  | 0.076 |
| Employed | 231 (91.3) |  | 159 (85.9) |  |  |
| Unemployed or >50% on sick leave | 22 (8.7) |  | 26 (14.1) |  |  |
| **Financial concerns** |  |  |  |  | 0.077 |
| No | 160 (63.7) |  | 103 (55.4) |  |  |
| Yes | 91 (36.3) |  | 83 (44.6) |  |  |
| **Smoking** |  |  |  |  | 0.863 |
| No | 221 (88.0) |  | 163 (88.6) |  |  |
| Yes, daily/occasionally | 30 (12.0) |  | 21 (11.4) |  |  |
| **Previous abdominal surgery** |  |  |  |  | 0.982 |
| No | 161 (63.9) |  | 118 (63.8) |  |  |
| Yes | 91 (36.1) |  | 67 (36.2) |  |  |
| **Preoperative pain** (any location) |  |  |  |  | <0.001 |
| No | 139 (55.2) |  | 51 (27.6) |  |  |
| Yes | 113 (44.8) |  | 134 (72.4) |  |  |
| **Average pain week pre-surgery*** (NRS 0-10) Median (IQR) |  | 2 (0-4) |  | 5 (2-6) | <0.001 |
| **Expected postoperative pain*** (NRS 0-10) Mean (SD) |  | 4.8 (2.1) |  | 6.1 (2.1) | <0.001 |
| **Worst menstrual pain*** (NRS 0-10)  Median (IQR) |  | 6 (0-8) |  | 8 (6.5-10) | <0.001 |
| **Preoperative non-opioid analgesics** |  |  |  |  | <0.001 |
| No | 61 (24.1) |  | 19 (10.3) |  |  |
| Yes | 192 (75.9) |  | 166 (89.7) |  |  |
| **Preoperative opioids** |  |  |  |  | <0.001 |
| Daily | 3 (1.2) |  | 1 (0.5) |  |  |
| Weekly | 9 (3.6) |  | 19 (10.3) |  |  |
| Less than weekly | 28 (11.3) |  | 41 (22.3) |  |  |
| Not applicable | 208 (83.9) |  | 123 (66.9) |  |  |

* = continuous variable

Abbreviations: SD, Standard Deviation; IQR, Interquartile Range; NRS, Numeric Rating Scale; BMI, Body Mass Index; ASA, American Society of Anesthesiologists

NRS is used as a continuous variable when comparing groups (Group NS vs. Group S).

Supplementary Table 2. Type of surgery, duration of surgery and anaesthesia, time spent in the PACU (n= 439).

| **Laparoscopic procedures** | **n (%)** |
| --- | --- |
| **Minor surgeries** |  |
| Ovarian cyst removal (uni- or bilateral) | 62 (14.1) |
| Salpingo-oophorectomy (uni- or bilateral) | 58 (13.2) |
| Salpingectomy (uni- or bilateral) | 16 (3.6) |
| Chromopertubation | 6 (1.4) |
| Ovarian drilling for polycystic ovary syndrome (uni- or bilateral) | 2 (0.5) |
| Diagnostic laparoscopy with no further surgery | 44 (10.0) |
| Oophorectomy (uni- or bilateral) | 1 (0.2) |
| Removal of migrated Intrauterine device | 8 (1.8) |
| Sterilisation/Tubal ligation | 25 (5.7) |
|  |  |
| **Major surgeries** |  |
| Excision of endometriosis | 211 (48.1) |
| Adhesion removal | 6 (1.4) |
|  |  |
| **Minor surgeries** | 222 (50.6) |
| **Major surgeries** | 217 (49.4) |
|  |  |
|  | **Median (IQR)** |
| **Duration of surgery (min)** | 38 (28-52) |
| **Duration of anaesthesia (min)** | 68 (57-85) |
| **Time spent in PACU (min)** | 171 (127-221) |
|  |  |

Abbreviations: PACU, Post Anaesthesia Care Unit; IQR, Interquartile Range

Supplementary Table 3. Postdischarge analgesia consumption until 24 h after end of surgery, for the whole sample (n= 439).

| **Standard postdischarge analgesia** | **Number of users n (%)** | **Median (IQR)** | **Min-Max**  **total dose** | **Comments** |
| --- | --- | --- | --- | --- |
| Paracetamol mean (SD) | 421 (95.9) | 2 g (1.2 g) | 0 -5 g | Over-the-counter medicine |
| Ibuprofenᵃ | 362 (82.5) | 1200 mg (400 - 1200 mg) | 0 - 2400 mg | Over-the-counter medicine |
| Immediate-release oxycodone | 240 (54,7) | 5 mg (0 - 10 mg) | 0 - 25 mg | In-house supply of four 5 mg capsules |
| Tramadol | 88 (20.0) | 0 mg (0 - 0 mg) | 0 - 250 mg | In-house supply of six 50 mg capsules |
| **Other types of analgesics used** |  |  |  |  |
| Modified-release oxycodone | 3 (0.7) | 0 mg (0 – 0 mg) | 0 – 10 mg | Two patients were admitted to hospital and received modified-release oxycodone during hospitalisation. One patient, discharged as planned, was provided with an in-house supply of modified-release oxycodone. |
| Codeine | 3 (0.7) | 0 mg (0 – 0 mg) | 0 – 30 mg | Private supply |
| Amitriptyline (Sarotex) | 1 (0.2)* | 0 mg (0 – 0 mg) | 0 – 30 mg | Patients’ regular medication (30 mg) |
| Pregabalin (Lyrica) | 1 (0.2)* | 0 mg (0 – 0 mg) | 0 – 450 mg | Patients’ regular medication (150 mg x 3) |
| Gabapentin (Neurontin) | 1 (0.2)* | 0 mg (0 – 0 mg) | 0 – 600 mg | Patients’ regular medication (600 mg) |

ᵃ After discharge, a few patients chose to use another type of NSAID than ibuprofen: four patients (0.9%) used Diclofenac (min-max total dose 50-200 mg), and six patients (1.4%) used Naproxen (min-max total dose 500-2000 mg).

* Not the same patient
